# Hydatid Cyst in Liver Masquerading As Metastatic Deposits From Ovarian Carcinoma

**DOI:** 10.7759/cureus.17503

**Published:** 2021-08-27

**Authors:** Vishwapriya M Godkhindi, Nitin Gupta, Ananth Pai, Afees Ahamed M A, Kavitha Saravu

**Affiliations:** 1 Department of Pathology, Kasturba Medical College, Manipal Academy of Higher Education, Manipal, IND; 2 Department of Infectious Diseases, Kasturba Medical College, Manipal Academy of Higher Education, Manipal, IND; 3 Department of Medical Oncology, Kasturba Medical College, Manipal Academy of Higher Education, Manipal, IND; 4 Department of Internal Medicine, Kasturba Medical College, Manipal Academy of Higher Education, Manipal, IND

**Keywords:** cystic echinococcosis, hepatic echinococcosis, echinococcus granulosus, hydatid disease, hydatid cyst

## Abstract

Metastatic deposits from primary ovarian malignancy can manifest as cystic masses in the liver. In endemic areas, hydatid disease is an important differential in all cases of cystic hepatic masses. We report a case of a 55-year-old lady who presented with progressive abdominal distension and was diagnosed with primary ovarian high-grade serous carcinoma. Imaging revealed concurrent lesions in the liver that were thought to be metastatic deposits but was later diagnosed as hydatid cyst based on radiologic features and serology. We report this rare case to highlight the importance of suspecting a hydatid cyst in endemic areas and its varied manifestations.

## Introduction

Hydatid cyst is a zoonotic disease caused by the larvae of *Echinococcus granulosus* [1]. The canines are the definitive host of this cestode while sheep are the intermediate host [1]. Humans are accidental hosts and may acquire the disease from ingesting food contaminated with the feces (containing eggs) of the definitive host. This disease is endemic in developing countries where sheep and dogs are raised together. The liver is the most common site of involvement, followed by the lungs [2]. It is usually asymptomatic and is detected incidentally in most cases [2]. It is important, however, to diagnose this early as the enlarging cyst is associated with a variety of complications including obstruction of the flow of blood/lymphatics and cyst rupture [3,4]. The hydatid cyst can be sometimes confused with metastasis from other organs [5]. We report one such case of hydatid cyst in the liver that was initially confused with metastasis from a primary ovarian carcinoma.

## Case presentation

**Figure 1:**
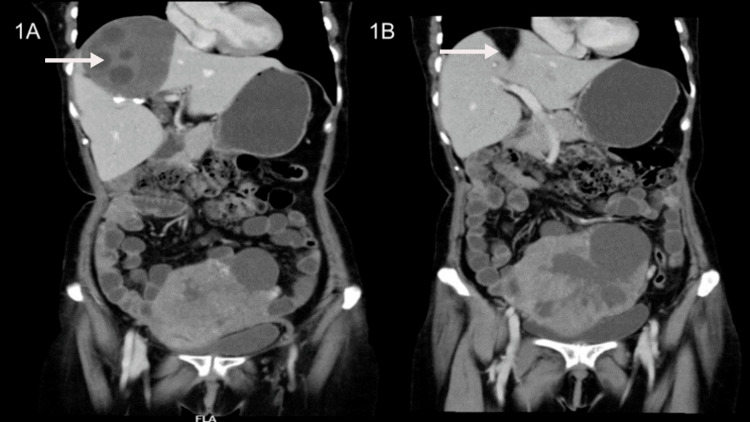
(A) Contrast-enhanced computed tomography of the abdomen and pelvis shows a complex solid-cystic enhancing lesion in the pelvis with an enhancing multicystic lesion in the liver. (B) (Post-chemotherapy) Contrast-enhanced computed tomography of the abdomen and pelvis shows a significant reduction in the size of the liver and ovarian lesion.

**Figure 2:**
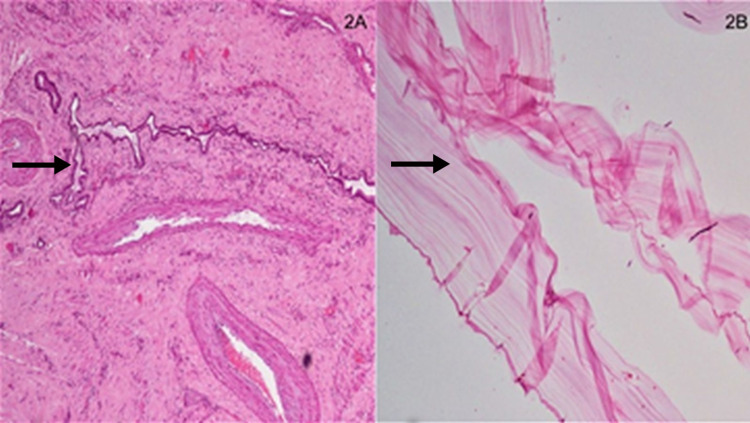
(A) Section shows the outermost pericyst with entrapped bile ductules and fibroblastic reaction (H&E 10x). (B) Section studied shows the acellular laminated membrane, a hallmark of hydatidosis (H&E 10x).

A 55-year-old lady presented with complaints of low-grade fever and dull-aching lower abdominal pain of six months duration. On physical examination, she was found to have hepatomegaly (up to 4 cm below right costal margin) and an abdominal mass (8 x 4 cm) in the hypogastrium. The pelvic mass had a firm consistency with side-to-side mobility and an impalpable lower border. A contrast-enhanced computed tomography (CECT) of the abdomen and pelvis revealed a well-defined enhancing complex solid-cystic mass in the pelvis (9 x 7.9 x 10.3 cm) and multiple cystic lesions in the liver with enhancing double-layered cyst-wall with basal hydatid matrix/sand (Figure 1A). Ultrasound (USG)-guided biopsy of the ovarian lesion confirmed the diagnosis of high-grade serous carcinoma. Serology for *Echinococcus* IgG ELISA affirmed the diagnosis of hydatid disease. On USG, the hydatid cyst was classified as type CE3b (multilocular cysts with daughter cyst in the solid matrix). She was treated with pre-operative PO albendazole (400 mg BD x 28 days) with concurrent three cycles of three-weekly neoadjuvant chemotherapy (paclitaxel with carboplatin) for high-grade serous ovarian carcinoma following which interval debulking surgery with hepatic hydatid cyst drainage and excision of the cyst was done. Histopathological examination of the hepatic cystic tissue specimen confirmed the diagnosis of hydatid cyst with the demonstration of outermost reactive pericyst and inner acellular laminated membrane, i.e., ectocyst (Figures 2A, 2B) and examination of the ovarian mass revealed residual viable high-grade serous carcinoma. Post-operatively, she received PO albendazole (400 mg BD) for two weeks with three more cycles of adjuvant chemotherapy (paclitaxel with carboplatin). A repeat CECT-abdomen and pelvis were done which showed complete resolution with fat replacement of the hydatid cyst in the liver (Figure 1B). She has completed her adjuvant chemotherapy and has recuperated well and is on regular follow-up for the last months.

## Discussion

Ovarian cancer is among the most common cancers in women with a considerable burden in developing countries like India [6]. Most of the malignant ovarian tumors are primary, while only a fraction of these represents secondary deposits from primaries in the gastrointestinal tract or breasts [7]. Most of the patients are diagnosed late, and only a few of the cases are confined to the ovaries at the time of diagnosis [8]. Most patients present with bloating, pelvic/abdominal pain, or urinary symptoms. The prognosis is good for women diagnosed at an early stage (confined to ovary or pelvis), whereas the majority, diagnosed at later stages (extending outside pelvis), are likely to survive less than five years [9].

Ovarian cancer can directly extend into the peritoneum or metastasize to the bowel, liver breast or spleen [9-11]. In this case, lesions in the liver were initially suspected to be metastatic deposits. Although the metastatic deposits in the liver from the ovary can be cystic due to the high fluid content, the lesions in the liver on CT scan, in this case, were more consistent with hydatid cysts as daughter cysts could be visualized [11]. This was confirmed with serological testing. The detection of IgG antibodies against *Echinococcus* spp. is used for the serological diagnosis of hydatid cyst. ELISA has good sensitivity for making a diagnosis of liver hydatid cyst [12] with IgG ELISA being more sensitive than IgM ELISA [13].

Although both USG and CECT-scan have good sensitivity for diagnosing hydatid cyst, USG is preferred for staging as it is cost-effective whereas CT is preferred for delineating the number, size, and location of the hepatic cyst [14,15]. USG is used to classify the lesions into active (CE1,2), transitional (CE3), or inactive (CE4,5) stages [16]. The USG findings of this patient corresponded to CE3b. Since CE3b cysts have many compartments, puncture aspiration injection and re-aspiration (PAIR) are not preferred. Patients in this stage are managed by a combination of surgery and drug therapy with albendazole [16].

## Conclusions

In summary, we report a case of an elderly woman who was diagnosed with primary ovarian malignancy and incidentally detected hepatic hydatid cyst masquerading as metastatic deposits. The clinical, radiological, and serologic evaluation helped clinch the diagnosis of hydatid cyst. We report this rare case to highlight the need for suspicion of hydatid cyst in patients from endemic areas in light of relevant radiological and serologic findings.
